# Neuroprotective effect of kinin B1 receptor activation in acute cerebral ischemia in diabetic mice

**DOI:** 10.1038/s41598-017-09721-0

**Published:** 2017-08-25

**Authors:** Dorinne Desposito, Georges Zadigue, Christopher Taveau, Clovis Adam, François Alhenc-Gelas, Nadine Bouby, Ronan Roussel

**Affiliations:** 1 INSERM U 1138, Cordeliers Research Center, Paris, France; 2 Paris Descartes University, Paris, France; 3 Pierre et Marie Curie University, Paris, France; 4 Anatomopathology Department, Kremlin-Bicêtre Hospital, Paris, France; 5 Denis Diderot University, Paris, France; 6 Diabetology, Endocrinology and Nutrition Department, DHU FIRE, Bichat Hospital, AP-HP, Paris, France

## Abstract

Activation of the kallikrein-kinin system enhances cardiac and renal tolerance to ischemia. Here we investigated the effects of selective agonists of kinin B1 or B2 receptor (R) in brain ischemia-reperfusion in diabetic and non-diabetic mice. The role of endogenous kinins was assessed in tissue kallikrein deficient mice (TK^−/−^). Mice underwent 60min-middle cerebral artery occlusion (MCAO), eight weeks after type 1-diabetes induction. Treatment with B1R-, B2R-agonist or saline was started at reperfusion. Neurological deficit (ND), infarct size (IS), brain water content (BWC) were measured at day 0, 1 and 2 after injury. MCAO induced exaggerated ND, mortality and IS in diabetic mice. B2R-agonist increased ND and mortality to 60% and 80% in non-diabetic and diabetic mice respectively, by mechanisms involving hemodynamic failure and renal insufficiency. TK^−/−^ mice displayed reduced ND and IS compared to wild-type littermate, consistent with suppression of B2R activity. B1R mRNA level increased in ischemic brain but B1R-agonist had no effect on ND, mortality or IS in non-diabetic mice. In contrast, in diabetic mice, B1R-agonist tested at two doses significantly reduced ND by 42–52% and IS by 66–71%, without effect on BWC or renal function. This suggests potential therapeutic interest of B1R agonism for cerebral protection in diabetes.

## Introduction

Acute brain ischemia secondary to cerebral artery occlusion is a major cause of mortality or permanent disability. Risk of ischemic stroke is increased in diabetic patients and prognosis is poorer^1, 2^. Cerebral artery occlusion causes acute (minutes to hours) and delayed (hours to days or weeks) injury cascades, both implicating multiple pathogenic factors like thrombosis, neuron stunning or necrosis, brain oedema and inflammation^3, 4^. The complexity of mechanisms involved in brain damage explains in part that there is still no clinically effective neuroprotective treatment besides revascularization. The kallikrein-kinin system (KKS) is implicated in physiological vasodilatation, exerts antithrombotic and profibrinolytic actions and reduces oxidative stress in different organs^5–8^. KKS protects against cardiac and renal damage in the setting of acute ischemia secondary to arterial occlusion. Inhibition of KKS aggravates cardiac and renal ischemic lesions while activation of kinin receptors enhances cardiac tolerance to ischemia and reperfusion^9–12^. Kinins are generated by proteolytic cleavage of protein precursors, kininogens, by tissue kallikrein (TK) and are mainly inactivated in the circulation by the angiotensin I-converting enzyme (ACE/kininase II)^5^. Kinins, activate two receptor subtypes: B1 (B1R) and B2 (B2R). All components of KKS have been identified in brain tissue from rodents and humans^3, 13–18^. B1R gene expression is low in the brain under normal condition, but it is upregulated by inflammation and ischemia^19, 20^. By contrast, B2R is constitutively present in different brain structures and in cerebral arteries and microvessels^18, 21^.

Role of kinins in brain ischemia has been addressed so far by performing pharmacological blockade of B1R or B2R in rodents or studying mice genetically deficient in either B1 or B2 receptor. However, these studies have produced conflicting results^3^. Some studies have shown that B2R blockade reduced infarct size and neuronal damage after transient middle cerebral artery occlusion (MCAO)^19, 22–24^ but other suggested that inactivation of this receptor has no effect or even aggravates ischemic brain damage^20, 25–27^. It has also been reported that pharmacological blockade or genetic inactivation of B1R confer neuroprotection in mice^20^. Single receptor inactivation however is well known to result in induction, coupling and activation of the remaining alternate receptor that can explain, at least in part, the effects observed making data interpretation ambiguous^8, 9, 28, 29^. Moreover, the effect of kinins and their receptors may depend in part on the stage of infarct development^27^. Therefore, the role of kinins, B1R and B2R in brain ischemia and the potential therapeutic interest of pharmacological manipulation of KKS need to be further documented by using new experimental approaches. Involvement of KKS in cerebral ischemia in the setting of diabetes has not been studied, except for a very recent report in the rat based on receptor inhibition^30^.

The aim of the present study was to address the role of KKS in cerebral ischemia in non-diabetic (NonDiab) and diabetic (Diab) mice, by using gain and loss of function approaches. We firstly probed the role of each receptor by performing pharmacological activation using potent, peptidase resistant synthetic agonists, selective B1R (B1R-ag) or B2R (B2R-ag) in a model of transient MCAO. We then addressed the role of endogenously produced kinins acting through both receptors by studying a genetic mouse model of TK and kinin deficiency.

The study shows that a) MCAO induced bradycardia, mild hypotension, neurological deficit, and resulted in partial brain infarction. Neurological deficit, mortality and infarct size were all increased in diabetic mice compared to non-diabetic mice; b) B2R activation increased neurological deficit and mortality; c) B1R activation had no effect in non-diabetic mice but in diabetic mice a B1R agonist, tested at two different dosages, reduced neurological deficit and infarct size; d) TK deficiency reduced neurological deficit and infarct size in non-diabetic mice but had no effect in diabetic mice. The data are consistent with a deleterious role of kinins, through B2R activation in cerebral ischemia. They however show that in diabetic condition B1R signalling is neuroprotective.

## Results

Effects of B1R or B2R agonists (ag) on cerebral ischemia were investigated, in both non-diabetic (NonDiab) and diabetic (Diab) mice. Diabetic mice were studied 8 weeks after streptozotocin injections. Focal cerebral ischemia (Isch) was induced *via* a transient intraluminal filament middle cerebral artery occlusion method. Non-ischemic (NonIsch) mice underwent sham operation. Chronic treatment with B1R-, B2R-agonist or saline was started at reperfusion, using osmotic minipumps implanted s.c. and lasted two days.

### Effect of ischemia on B1R and B2R mRNA levels

**Figure 1:**
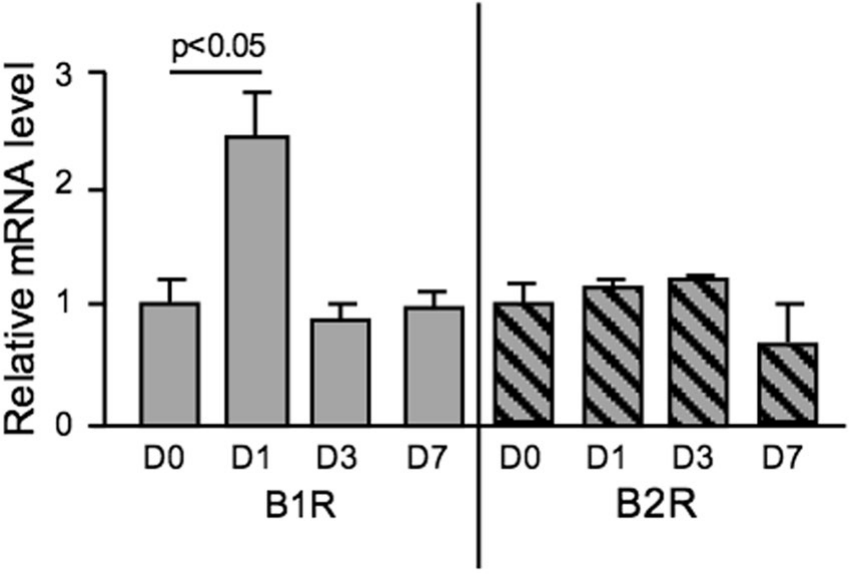
B1R mRNA level in brain increased 24 h after transient MCAO. Kinin receptor mRNA levels in NonDiab mice brain measured at day 0 (D0), 1 (D1), 3 (D3) and 7 (D7) after transient MCAO, by RT-qPCR. Data were normalized to 18 S rRNA. Values are mean ± SEM, n = 5/group.

B1R and B2R mRNAs were both detected in affected brain tissue. B2R mRNA level was not influenced by transient MCAO and did not change at day 1, 3 and 7 after ischemia in our model (Fig. 1). By contrast B1R mRNA level increased 2.35 fold (p < 0.05) 24 h after transient MCAO before returning to basal values (Fig. 1). B1R and B2R mRNA levels were not influenced by diabetes (data not shown).

### Effect of transient cerebral ischemia on neurological score and infarct size

**Figure 2:**
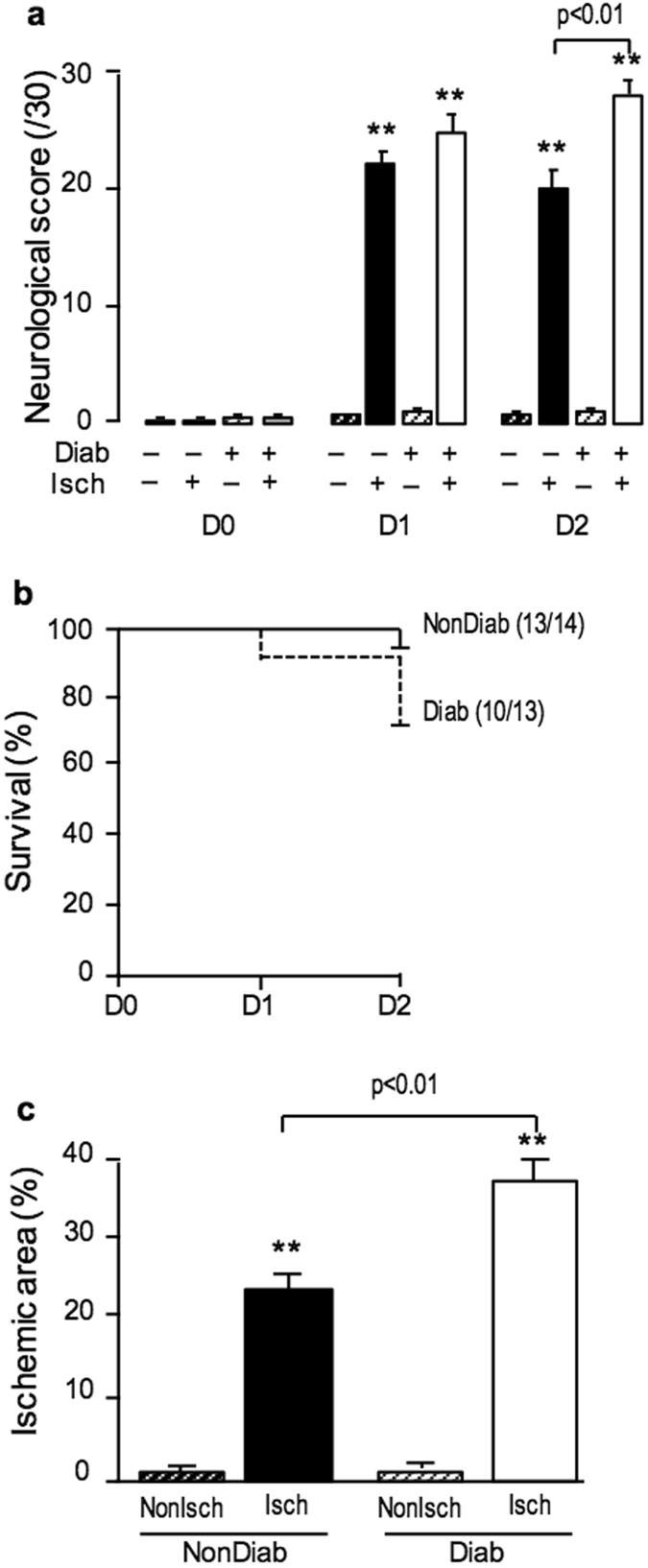
Diabetes increased neurological impairment, mortality and infarct size 48 h after transient MCAO. (**a**) Neurological score (0–30) measured in NonDiab and Diab mice at day 0, 1 and 2 after transient MCAO or sham-operation. (**b**) Survival curve of NonDiab and Diab mice after transient MCAO. Numbers in parentheses refer to surviving/operated animals. (**c**) Ischemic area measured at 48 h after transient MCAO or sham-operation using TTC staining. Values are mean ± SEM, n = 8–14/group. **p < 0.01 vs corresponding non ischemic group, other statistics shown on figure.

Transient MCAO did not affect body weight, glycaemia and plasma creatinine in either NonDiab or Diab mice (data not shown). In NonDiab mice, transient MCAO induced bradycardia (Isch: 382 ± 15 bpm *vs* NonIsch: 655 ± 14 bpm, *p* < 0.01) and a tendency to hypotension (Isch: 101 ± 2 mmHg *vs* NonIsch: 113 ± 2 mmHg, *p = *0.055), measured at 24 h. At 24 and 48 h after transient MCAO, the neurological score reflected severe impairment of sensorimotor function in Isch mice *versus* NonIsch mice (*p* < 0.01) (Fig. 2a). Infarction was observed in cerebral cortex and striatum (TTC staining: 24.1 ± 2.0%, *p* < 0.01 *vs* NonIsch). Mortality remained low (Fig. 2b).

Diabetes increased neurological deficits (p < 0.01) at 48 h when compared to NonDiab mice (Fig. 2a,b). Infarct size was increased by 55% in Diab mice compared to NonDiab mice (TTC staining: 37.2 ± 2.7 *vs* 24.0 ± 2.8%, *p* < 0.01, Fig. 2c).

### Effect of B2R agonist treatment after transient cerebral ischemia

**Figure 3:**
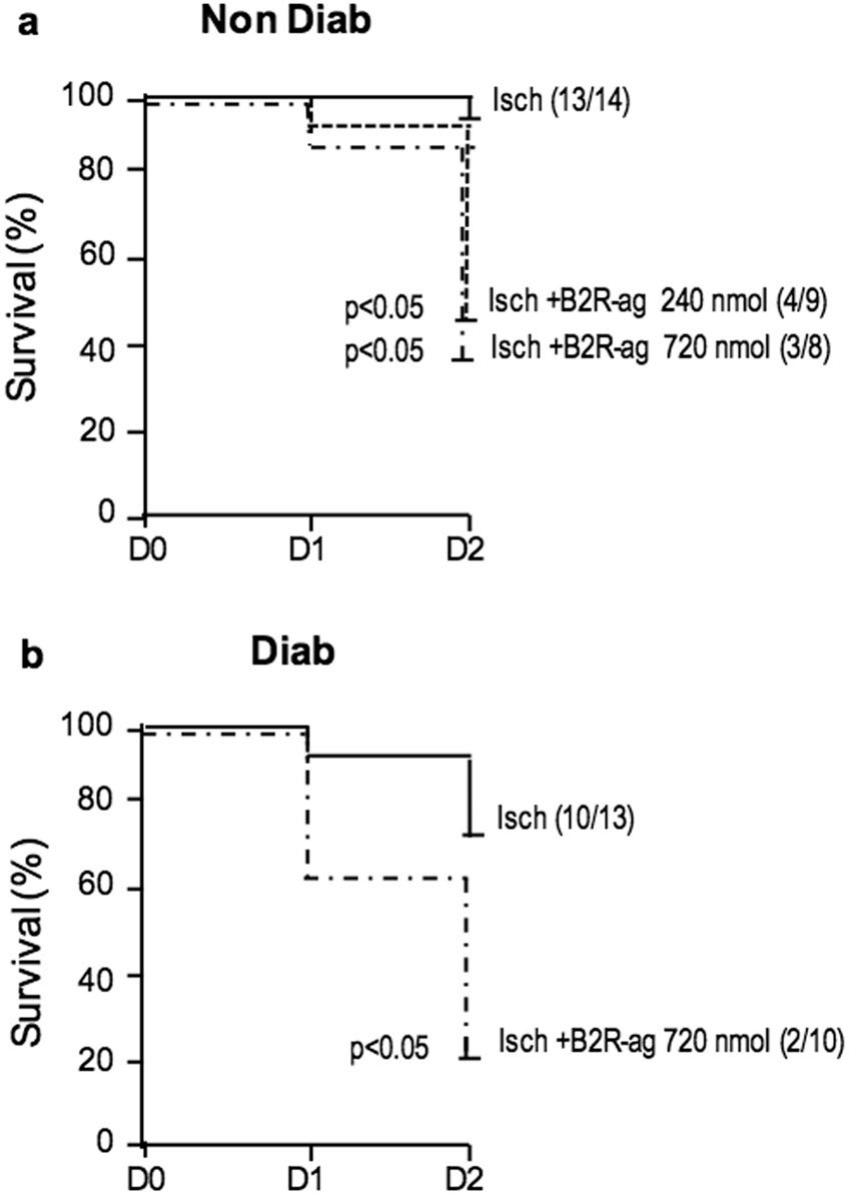
B2R-ag treatment was associated with increased mortality after transient MCAO. (**a**) Survival curve of NonDiab mice treated with B2R-ag (720 or 240 nmol/kg.day^−1^) or non-treated (Isch). (**b**) Survival curve of Diab mice treated with B2R-ag (720 nmol/kg.day^−1^) or non-treated (Diab-Isch). Numbers in parentheses refer to surviving/operated animals. Values are mean ± SEM, n = 8–14/group. p < 0.05 refers to corresponding ischemic group.

B2R-ag treatment significantly increased mortality to 60% in NonDiab mice and 80% in Diab mice 48 h after ischemia (both *p* < 0.05 compared to saline, Fig. 3). Mortality occurred mainly after 24 h. This did not allow gathering enough data concerning neurological deficits and histological lesions at two days for these groups.

In NonDiab ischemic mice, B2R-ag treatment did not influence bradychardia (Isch: 382 ± 15 bpm, Isch + B2R-ag 720 nmol/kg.day^−1^: 460 ± 17 bpm, Isch + B2R-ag 240 nmol/kg.day^−1^: 404 ± 20 bpm, both NS) and aggravated hypotension at the two different dosages used (Isch: 101 ± 2 mmHg, Isch + B2R-ag 720 nmol/kg.day^−1^: 87 ± 2 mmHg, Isch + B2R-ag 240 nmol/kg.day^−1^: 82 ± 6 mmHg, both *p* < 0.01 vs NonIsch), measured at day 1 after MCAO. The treatment severely increased plasma creatinine, in surviving animals at day 2 (Isch: 17.5 ± 4.2 μmol/l *vs* Isch + B2R-ag 720 nmol/kg.day^−1^: 72.8 ± 19.1μmol/l, *p* < 0.05). B2R-ag treatment did not affect body weight and glycaemia at the two dosages (data not shown). In NonIsch mice, B2R-ag treatment did not influence blood pressure (NonIsch + B2R-ag: 102 ± 2 mmHg, NS), heart rate (667 ± 23 bpm, NS) or plasma creatinine (NonIsch + B2R-ag: 15.4 ± 0.5 μmol/l, NS).

### Effect of B1R agonist treatment in mice after transient cerebral ischemia

**Figure 4:**
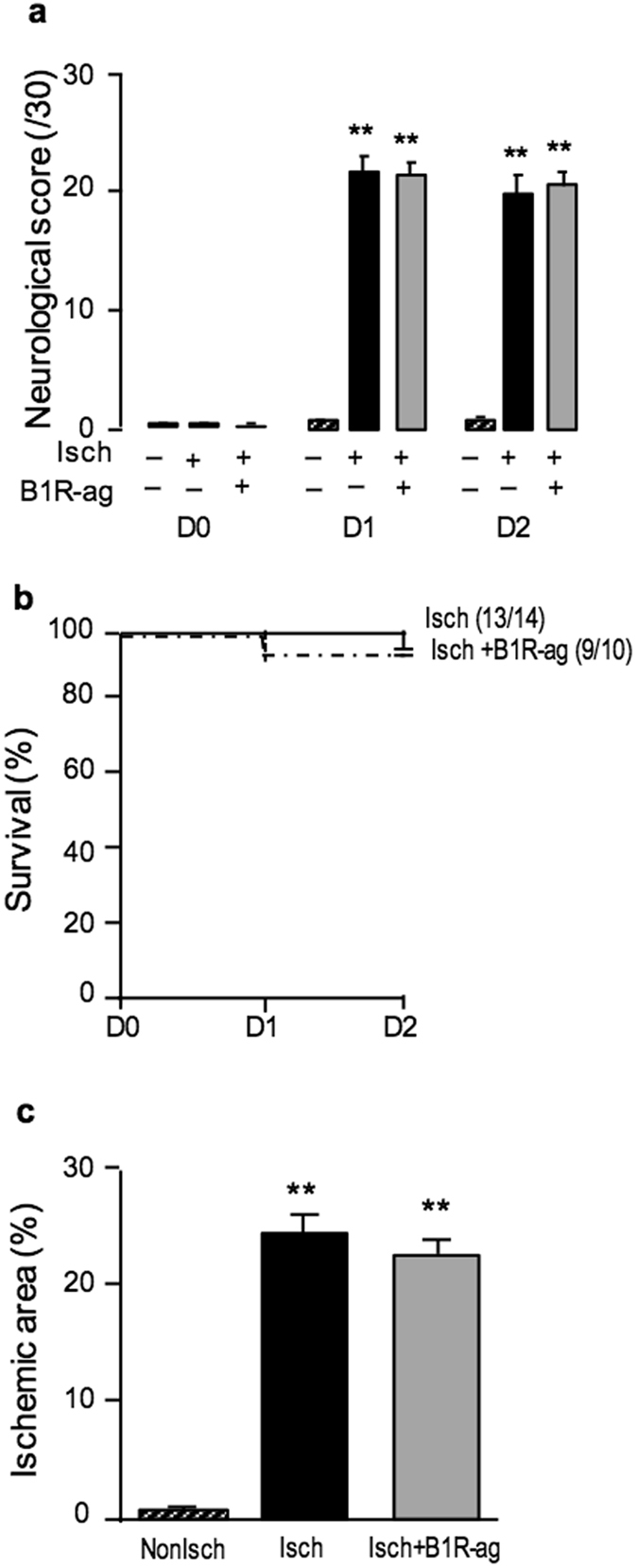
B1R-ag had no effect on neurological impairment, mortality and infarct size 48 h after transient MCAO in NonDiab mice. (**a**) Neurological score (0–30) measured in NonDiab mice, treated with B1R-ag (720 nmol/kg.day^−1^) (grey bars) or saline (black bars), at day 0, 1 and 2 after transient MCAO or sham-operation. (**b**) Survival curve of NonDiab mice treated with B1R-ag or non-treated after transient MCAO. Numbers in parentheses refer to surviving/operated animals. (**c**) Ischemic area measured at 48 h after transient MCAO using TTC staining. Values are mean ± SEM, n = 9–14/group. **p < 0.01 vs non ischemic group.

Neurological deficit and mortality was not influenced by B1R-ag treatment (720 nmol/kg.day^−1^) in NonDiab mice (Fig. 4a,b). Treatment had no effect on body weight, glycaemia, plasma creatinine, blood pressure and heart rate when compared to saline (data not shown). B1R-ag treatment had no effect on infarct volume and histological score at day 2 after transient MCAO (Fig. 4c).

**Figure 5:**
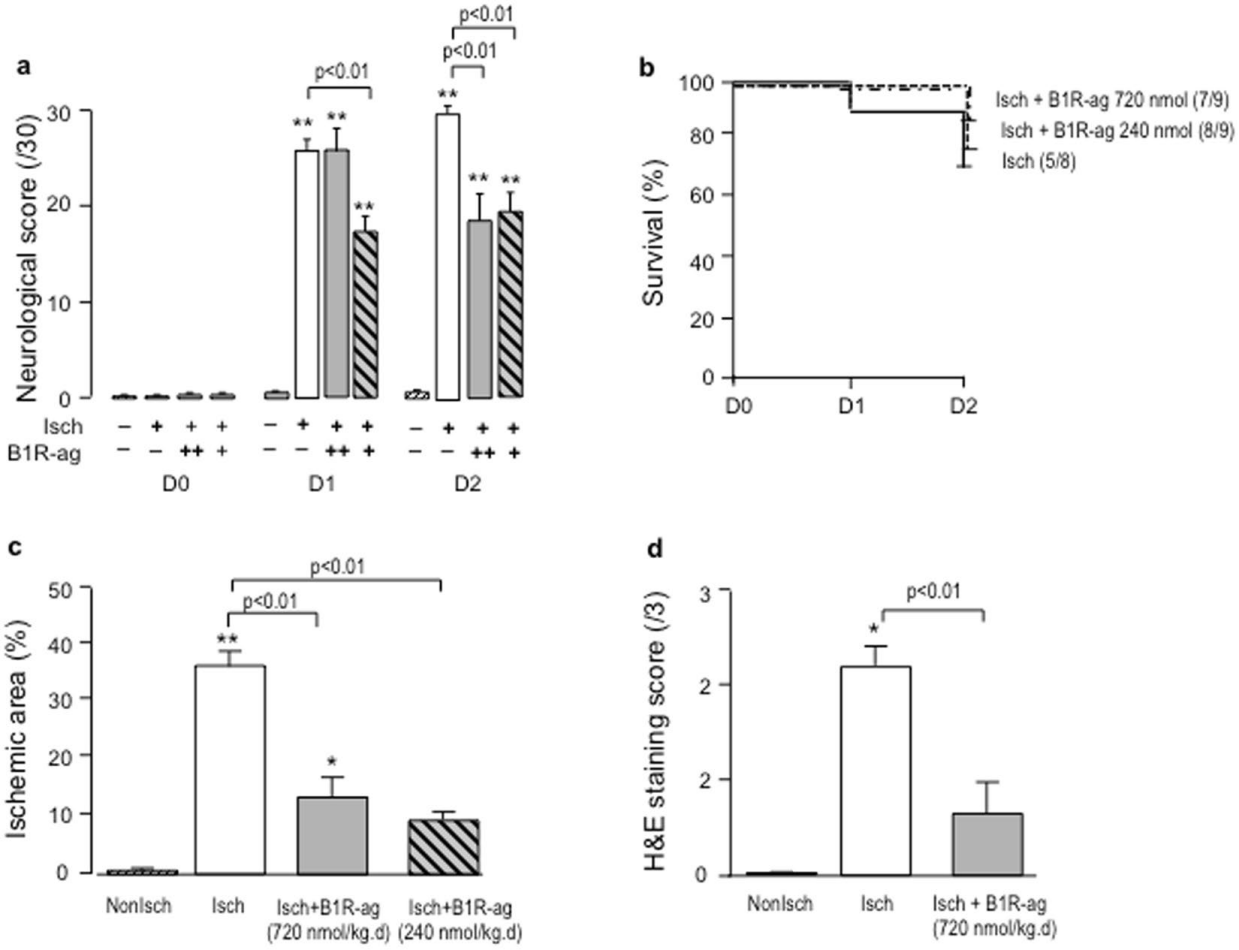
B1R-ag, at two different dosages (720 or 240 nmol/kg.day^−1^), reduced neurological score and infarct size at 48 h in Diab mice. (**a**) Neurological score (0–30) measured in Diab mice treated with B1R-ag, 720 nmol/kg.day (grey bars) or 240 nmol/kg.day^−1^ (hatched bars) or with saline (white bars), at day 0, 1 and 2 after transient MCAO. (**b**) Survival curve of Diab mice after transient MCAO. Numbers in parentheses refer to surviving/operated animals. (**c**) Ischemic area measured at 48 h after transient MCAO using TTC staining. (**d**) Histological score (0–3) measured at 48 h after transient MCAO using haematoxylin and eosin (H&E) staining. Values are mean ± SEM, n = 6–13/group. *p < 0.05, **p < 0.01 vs non ischemic group. Other statistics shown on figure.

By contrast, in Diab mice, B1R-ag tested at two different dosages (240 or 720 nmol/kg.day^−1^) improved neurological score compared to saline treated Diab-Isch group (Fig. 5a,b). The clinical beneficial effect of B1R-ag was associated with a decrease of infarct size by 71 and 66% at the two dosages respectively (both p < 0.01) (Fig. 5c). These results were confirmed by the histological score (Fig. 5d). B1R-ag did not induce mortality (Fig. 5b) and did not increase plasma creatinine (Diab-Isch: 17.3 ± 0.3 μmol/l vs Diab-Isch + B1R-ag: 16.7 ± 1.1 μmol/l, NS). Moreover, B1R-ag at 720 nmol/kg.day^−1^ did not affect BWC 24 h after cerebral ischemia-reperfusion compared to saline-treated Diab-Isch group (Diab-Isch: 83.2 ± 0.82% *vs* Diab-Isch + B1R-ag: 82.3 ± 0.27%, NS).

### Effect of TK deficiency on transient cerebral ischemia

**Figure 6:**
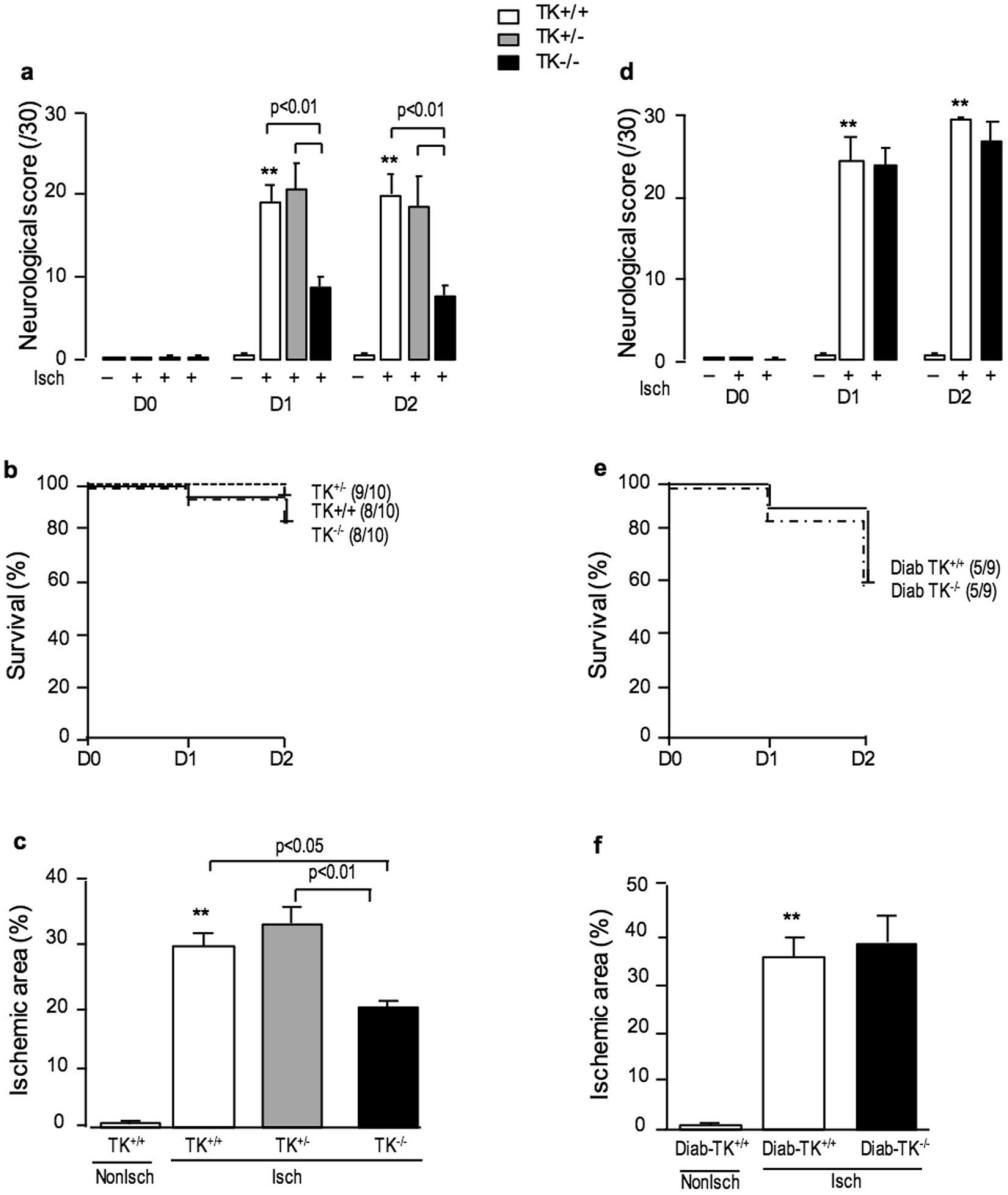
TK deficiency decreased neurological impairment and infarct size 48 h after transient MCAO in NonDiab mice but had no effect in Diab mice. (**a**,**d**) Neurological score (0–30) measured in TK-deficient mice at day 0, 1 and 2 after transient MCAO. (**b**,**e**) Survival curve of TK-deficient mice after transient MCAO. Numbers in parentheses refer to surviving/operated animals. (**c**,**f**) Ischemic area measured at 48 h after transient MCAO using TTC staining. a, b and c: NonDiab mice; d, e and f: Diab mice. TK^+/+^: white bars, TK^+/−^: grey bars, TK^−/−^: black bars. Data are mean ± SEM, n = 8–10/group. **p < 0.01 vs non ischemic group. Other statistics shown on figure.

In NonDiab condition, TK^−/−^ mice displayed better post MCAO outcome compared to TK^+/+^ and TK^+/−^ mice. Indeed, TK deficiency improved neurological score and significantly decreased infarct size at 48 hours after transient MCAO (Fig. 6a–c). In Diab mice however, TK deficiency had no effect on cerebral ischemia-reperfusion outcome (Fig. 6d–f).

## Discussion

In the present study, we evaluated effect of kinin signalling in mice submitted to transient focal cerebral ischemia. We studied both diabetic and non-diabetic mice because of the deleterious effect of diabetes on outcome of cerebral ischemia in man and also of diabetes effect on cellular signalling pathways^2, 11^. We considered the two kinin receptor subtypes, B1 or B2 and probed their effects by selectively activating these receptors, pharmacologically. We then addressed the role of endogenously produced kinins by studying mice deficient in TK, the main kinin-forming enzyme. We show that B2R activation increases mortality after transient MCAO in non-diabetic or diabetic mice, by mechanisms that may involve peripheral hemodynamic failure. B1R signalling on the other hand is not detrimental and its effect is strongly influenced by diabetes. While B1R activation has no effect in non-diabetic mice it reduces brain infarction and improves MCAO outcome in diabetic mice. Data obtained in TK deficient mice are consistent with disappearance of B1 and B2 receptors activity and suggest a role of endogenously produced kinins in cerebral tolerance to ischemia. Finding that treatment with a selective B1R agonist, at different dosages, reduces brain infarct volume and improve neurological deficit in diabetic mice may have therapeutic implication.

Previous studies indicated that B1R and B2R are present in the brain from various species including man^3, 13, 14, 16–18^. We show that genes for both receptors are expressed in murine brain and their expression level is not altered by diabetes. Whereas B2R mRNA was not influenced by ischemia, B1R mRNA level increased in the ischemic hemisphere after transient MCAO. This is consistent with previous studies^19, 20^ and extends to the brain observation of induction of B1R gene expression by ischemia made in the heart and kidney^9, 10, 31^. Increase in B1R mRNA in the ischemic brain was transient, peaking at 24 h and then subsiding, despite brain infarction. This is similar to what has been observed in the ischemic heart suggesting that acute phase secretion of proinflammatory cytokines acting through MAP-kinases and NFkappaB activation but not post-necrosis tissue remodelling and fibrosis development is involved in B1R induction in the ischemic brain^31, 32^.

Transient MCAO resulted in downstream brain infarction and, clinically, severe neurological impairment. Bradychardia is believed to result from reflex activation of baroreflex loop^33^ and may contribute to hemodynamic instability with mild decrease in blood pressure. Activation of B2R signalling pathways during reperfusion induced mortality. While transient MCAO did not result in significant mortality, more than 50% of B2R agonist treated mice died within two days, mainly after one day. Analysis of the brain in surviving animals did not show larger brain infarction or evidence for aggravated cerebral oedema, despite known effects of kinins on cerebral oedema^19, 34^, compared to saline treated animals (data not shown). However, these observations are difficult interpreting in term of causality, or lack of it, between B2R activity and brain damage and no conclusion can be made in absence of data concerning brains of deceased animals. Data obtained in TK deficient mice however indirectly suggest that B2R activity may aggravate brain infarction (see below). The cause of death of B2R agonist treated animals remains undocumented but may be related, at least in part to peripheral hemodynamic failure. These animals indeed displayed aggravated hypotension when compared to saline treated animals and had severe renal insufficiency. These two effects were not observed during B2R agonist treatment at the same dosage and by the same route in mice non-submitted to cerebral ischemia or in other experimental settings, including in diabetes, ruling out renal toxicity of the B2R agonist^12, 35^ (unreported data). Thus the effect of B2R activation on blood pressure, renal function and mortality in cerebral ischemia-reperfusion is peculiar to this experimental setting. One can speculate that in presence of inappropriate baroreflex activation triggered by brain ischemia B2R agonist administration induces hypotension that may, if becoming severe, even transiently, result in renal failure and death. This phenomenon would likely not occur in man where sympathetic activation raising blood pressure is a major effect of acute brain ischemia^36^.

B1R activation had no effect on mortality and brain infarction in non-diabetic animals. Interestingly, TK deficiency reduced infarct size and improved neurological defects in the non-diabetic animals. This result suggests that endogenously produced kinins are involved in brain damage during ischemia-reperfusion. Data obtained with subtype selective pharmacological receptor agonists suggest that effect of endogenous kinins can be ascertained to B2R but not B1R activation.

Diabetes increased infarct size and enhanced neurological impairment, consistent with clinical studies. The effect of the kallikrein-kinins system in brain ischemia in the setting of diabetes has not been studied until now. Like in non-diabetic animals B2R activation enhances mortality. But interestingly, a B1R agonist, when administered at time of reperfusion, improves neurological deficit and decreases brain infarct size by more than 60% in diabetic mice submitted to transient focal cerebral ischemia. No adverse effect on mortality or renal function was observed during B1R agonist treatment. Thus, in diabetic mice B1R activation has neuroprotective effect in cerebral ischemia. Mechanisms remain unclear but may be related to endothelial activation with release of anticlotting, profibrinolytic and vasodilatory agents^37–39^. Vasodilatation of collateral arteries could improve cerebral blood supply. Also, kinins are known to modulate mitochondrial permeability transition pore opening and trigger production of reactive oxygen species, which afford organ protection^7, 40, 41^. These mechanisms are believed to be operative in the ischemic heart or kidney^10, 11^. The lack of effect of B1R agonist on brain infarction in non-diabetic animals may appear surprising but the situation is similar to the ischemic heart where B1R activation had no effect in non-diabetic mice while it dramatically reduced infract size in diabetic animals^11^. Hypothesis put forward for the heart and kidney and based on the well documented balance between B1R and B2R activity^8, 9, 28^ may also be valid for the brain: when B2R is functional in non-diabetic animals B1R remains uncoupled. However, in the ischemic heart and tentatively brain of diabetic animals, B2R signalling is impaired and B1R coupling is activated^11^. Effect of B2R on mortality in diabetic animals may be due to peripheral rather than cerebral action, as discussed above. A recent report by Sang *et al*.^30^ suggests that acute administration of a B1R antagonist in type 2 diabetic rats submitted to MCAO reduces brain infarction. Our data may not appear consistent with this observation. However, result of the Sang *et al*. study is difficult interpreting given that the compound used as B1R antagonist is an analog of human rather than rat kinins^42–45^ and can behave as a partial agonist instead, depending on kinin level.

TK deficiency in diabetic mice did not influence MCAO outcome suggesting that TK is not involved in kinin production in brain of diabetic animals. An alternate explanation is that the lack of effect of TK deficiency results from disappearance of both the beneficial effect of B1R activation and the deleterious effect of B2R activation.

Our study clarifies to some extent the controversial issue of role of kinin and their receptors in cerebral ischemia-reperfusion. The study show that B2R activation is detrimental in this experimental setting but B1R activation can be beneficial. Documentation of neuroprotective effect of a pharmacological B1R agonist in brain ischemia in diabetic mice can have therapeutic implication. Together with previous studies documenting cardioprotective effect of the B1R agonist in the diabetic and ischemic mouse heart^11^ and proangiogenic effect in peripheral ischemia in diabetic mice^12^, the present observations argue for clinical development of kinin B1R agonist for cardiovascular and cerebral protection in diabetes.

## Materials and Methods

### Animals

Experiments were performed in male C57/BL6 mice (JanvierLabs, France or in house strain). TK deficient mice were generated in our laboratory by disruption of the TK gene as previously described^46^. Littermate wild type, homozygous and heterozygous TK deficient mice were obtained by heterozygous crossing^46, 47^. All mice were housed with a 12 h light/dark cycle and had free access to standard mice chow and water. All experimental procedures were performed in accordance with the Directive 2010/63/eu of the European Union. The study has received approval from the Ethical Committee Charles Darwin (CEEACD/N°5). Reporting of this work complies with ARRIVE guidelines.

### Murine model of type 1 diabetes

Diabetes (Diab) was induced in ten weeks-old mice by 5 daily i.p. injections of streptozotocin (STZ) (Sigma-Aldrich, France) (50 mg/kg body weight in 0.05 mol/L sodium citrate, pH 4.5)^12^. After 8 weeks of established diabetes (fasting blood glucose >250 mg/dl), transient focal cerebral ischemia was induced as described below.

### Transient focal cerebral ischemia

Transient focal cerebral ischemia was induced by MCAO using the intraluminal filament technique previously described^48^. Briefly, mice were anesthetised with 3.5% isoflurane in an anaesthetic chamber and maintained during surgery at 2% isoflurane using a rodent mask. Body temperature was maintained at 37 ± 0.5 °C with a heating blanket throughout the entire experimental procedure. MCAO was carried out for 60 min by inserting a calibrated monofilament (Doccol Corporation, USA) according to body weight *via* the right external carotid artery into the internal carotid artery to block the origin of the MCA. Sham-operated controls (NonIsch) were treated similarly to the ischemic (Isch) mice, but the filament was not inserted. After surgery and before being returned to cages, animals were placed for 4 hours in a heating incubator at 37 °C.

### B1R or B2R agonist treatments

Chronic treatment with the selective B1R agonist SarLys[Hyp3, Igl5, DPhe8]desArg9-bradykinin (B1R-ag) or the selective B2R agonist [Hyp(3),Thi(5),(N)Chg(7),Thi(8)]-bradykinin (B2R-ag)^49, 50^ was started at reperfusion, using osmotic minipumps implanted s.c. (Alzet 1007D, Charles River Laboratories, France). These compounds are resistant to kininase hydrolysis. Two different dosages chosen from previous studies based on therapeutic efficiency and lack of hypotensive effect, 720 nmol/kg.day and 240 nmol/kg.day were used^12, 35^. Control mice received saline infusion.

### Experimental groups

Several sets of experiments were performed to analyse the effects of kinin receptor agonist treatments on mortality, neurological deficit and infarction volume (n = 8–14/group). All mice were ≈18 week-old at the time of MCAO. Animals were sacrificed after two days, unless otherwise indicated. Series 1 was dedicated at comparing effect of ischemia-reperfusion in NonDiab and Diab mice. Series 2 and 3 were dedicated at testing effect of B2R agonist at two dosages in NonDiab and Diab mice, respectively. Series 4 and 5 were dedicated at testing effect of B1R agonist at two dosages in NonDiab and Diab mice, respectively. The effect of B1R-ag (720 nmol/kg.day^−1^) on cerebral oedema in Diab mice 24 h after transient MCAO was tested in a separate series (n = 5–6/group).

Additional groups of NonDiab mice were dedicated at studying effect of B2R-ag (240 nmol/kg.day^−1^ or 720 nmol/kg.day^−1^) on blood pressure. Mice were treated for 24 h after transient MCAO. Neurological score was determined, blood pressure and heart rate was recorded, and animals were sacrificed (n = 5–6/group).

Other groups of mice rendered or not diabetic and submitted to MCAO occlusion or sham operation were used for studying time related effect of brain ischemia-reperfusion on receptor gene expression. Animals were sacrificed at 1, 3 or 7 days after surgery, brain was sampled and kept at −80% until processed for measurement of receptor mRNAs by RT-PCR (n = 5 per group and time point).

The effect of TK deficiency on neurological deficit and ischemic volume was tested in NonDiab mice at 48 h after transient MCAO, in TK^+/+^, TK^+/−^ and TK^−/−^ mice (n = 8–10/group). Same protocol was performed in series dedicated at testing effect of TK deficiency in Diab mice (Diab-TK^+/+^, Diab- TK^−/−^, n = 8–9/group).

### Measurement of blood pressure

Blood pressure was measured by tail-cuff plethysmography (BP-2000 Series II, BIOSEB Instruments, France) in trained animals as previously described^51^.

### Measurement of plasma creatinine

Plasma creatinine was assessed in blood samples taken at sacrifice using a colorimetric enzymatic assay (automatic analyser Konelab 201, France) (n = 4–5/group).

### Evaluation of neurological deficits

Neurological deficit was assessed in each animal on a numerical scale of 0–30 before ischemia and at day 1 and/or 2 after ischemia, depending on protocol. The score was obtained by using a series of behavioural tests including grip test, scotch test, tail suspension test, beam test, wire hang test, circles tests and by assessing comportment into home cage as previously described^52–54^. Higher score indicate greater functional impairment. Mice were studied in random order in each series and blindly with regard to treatment.

### Determination of infarct volume

Two days after reperfusion and after neurological score evaluation mice were sacrificed. Brains were rapidly removed and sectioned into six coronal sections, 2 mm thick, using a mice brain matrix. Coronal brain sections were stained by incubation in a 0.5% 2,3,5-triphenyltetrazolium chloride (TTC, Sigma-Aldrich, France) solution for 30 min at 37 °C in the dark^55^ and fixed in 10% formalin (Sigma-Aldrich, France) for two hours prior to analysis. Photographs of the sections were obtained using digital camera attached to microscope (Nikon SMZ800, Italy). The infarction area, outlined in white, and the entire section area were measured under microscope (Nikon SMZ800, Italy) on the anterior surface of each section in a blinded manner with regard to protocol and treatment, using Image Analyzer Software (ImageJ, NIH). For each section, infarction area was normalized to the whole section area. For each animal, results for six sections were averaged.

Cerebral infarction was also evaluated by using Haematoxylin and eosin staining on the same sections of the brain for confirmation^56^. Brain sections were fixed in 10% formalin during 24 h, embedded in paraffin, cut into 6-μm section and stained with haematoxylin and eosin according to the manufacturer’s instructions (Sigma-Aldrich, France). Photomicrographs were obtained using digital camera attached to light microscope (Leica DM 4000B and LAS v3.8 software). Histological lesions were assessed in a blinded manner regarding protocol and treatment using a numerical scale of 0–3 for each animal^57^. Higher score indicate more severe histological lesions.

### Evaluation of brain oedema by measurement of brain water content (BWC)

Procedure was performed as previously described^58, 59^. Briefly, mice were killed by decapitation and brains were removed. Ischemic hemisphere was weighed before (wet weight) and after being dried at 110 °C for 24 h (dry weight). BWC was calculated and expressed as follows: BWC (%) = (wet weight − dry weight)/wet weight ×100.

### Quantification of B1R and B2R mRNA by real-time PCR

Total RNA was isolated from the whole ipsilateral hemisphere, in occluded or sham operated mice (day 0, 1, 3 and 7), using TRIzol (Invitrogen, France) and reverse transcribed with superscript II reverse transcriptase. The cDNAs were amplified and quantified using TaqMan Universal Master Mix and Assays-on-Demand Gene Expression Probes for gene of B1R and B2R (Applied Biosystems, France) in an ABI PRISM-7000 Sequence Detection System (Applied Biosystems, France), as previously described^60^. Each sample was tested in triplicate. Data were normalized to 18 S rRNA. Changes in the target gene were calculated by the 2^−∆∆CT^ comparative method for each sample^61^.

### Data expression and statistical analysis

Data are expressed as mean ± SEM. Effects of surgical procedure, diabetes and treatments on mortality and infarct size were evaluated by chi2 test or one-way ANOVA. For repeated measurements of neurological score two-way ANOVA was used. ANOVA was followed by Tukey multiple comparison tests. For comparison of mRNA level, data were analysed by Mann-Whitney test. Statistical significance was accepted at *p-value* less than 0.05.

### Data availability statement

The datasets generated during and/or analysed during the current study are available from the corresponding author on reasonable request.
